# Prolonged Length of Stay After Lung Resection: A Prediction Model That Loses Accuracy Across Hospitals, with No Added Benefit from Blood Pressure Data

**DOI:** 10.3390/jcm15176887

**Published:** 2026-09-05

**Authors:** Yuyao Zhu, Sunmian Xu, Cheng Li, Hai Chen, Jingxiang Wu

**Affiliations:** 1 Department of Anesthesiology, Shanghai Chest Hospital, Shanghai Jiao Tong University School of Medicine, 241 West Huaihai Road, Xuhui District, Shanghai 200030, China; zhuyuyao2022@163.com (Y.Z.); xusunmian@163.com (S.X.); 18298263458@163.com (C.L.); 2 School of Medicine, Tongji University, Shanghai 200331, China; chenhai424@163.com

**Keywords:** lung resection, prolonged postoperative length of stay, prediction model, intraoperative hypotension, temporal validation, external validation

## Abstract

**Background/Objectives**: It is uncertain whether intraoperative blood pressure (BP) summaries improve transportable prediction of prolonged postoperative length of stay (PLOS) after lung resection. We developed and temporally validated baseline and BP-enhanced models and evaluated their unchanged performance in the Korean VitalDB cohort. **Methods**: This retrospective prediction-model study used 536 local patients (249 PLOS events) for development and 184 later patients (74 events) for temporal validation. External validation included 626 procedure-restricted VitalDB patients; paired model comparisons used 624 patients with all four BP features. PLOS was a prespecified operational outcome defined as postoperative stay > 4 days. L2-penalized logistic models were assessed using nested cross-validation, discrimination, calibration, Brier score, and decision-curve analysis. **Results**: Baseline-model AUROCs were 0.714 (95% confidence interval [CI], 0.667–0.756) internally, 0.719 (0.640–0.788) temporally, and 0.623 (0.579–0.666) externally. Corresponding BP-enhanced AUROCs were 0.721, 0.725, and 0.615. BP features changed temporal AUROC by +0.006 (95% CI, −0.009 to +0.021) and external AUROC by −0.007 (−0.022 to +0.008). Temporal calibration slopes were 1.113 and 1.112, whereas external slopes were 0.570 and 0.534. Sensitivity analyses showed no consistent BP increment. **Conclusions**: The baseline model retained similar discrimination locally but had limited unchanged external transportability. Four intraoperative BP summaries provided no reproducible incremental predictive value. Neither model is ready for direct cross-center implementation without harmonization, updating, and further validation.

## 1. Introduction

Postoperative length of stay is a clinically important outcome after lung resection. A prolonged stay may reflect slower functional recovery, postoperative complications, or barriers within discharge pathways, and it influences patient counseling, bed allocation, and enhanced-recovery planning [1,2]. Earlier thoracic-surgery models have shown that prolonged hospitalization can be predicted using combinations of demographic, comorbidity, pulmonary, procedural, and perioperative factors [3,4,5]. However, many published models have been evaluated only within the datasets in which they were developed, and their transportability across institutions or countries remains uncertain [6].

Intraoperative hypotension is common during noncardiac surgery. Both the depth and duration of low mean arterial pressure (MAP) have been associated with postoperative organ injury and mortality in large observational cohorts [7,8,9]. Consensus recommendations therefore emphasize avoiding sustained MAP below approximately 60–70 mmHg while recognizing that optimal pressure may vary among patients [10]. Definitions remain heterogeneous across studies, particularly with respect to threshold, duration, and episode construction [11]. Continuous perioperative databases now permit BP exposure to be summarized by duration, area below a threshold, time-weighted average (TWA), and episode characteristics rather than by a single minimum value [9,12]. It is less clear whether these summaries improve prediction of recovery-oriented outcomes such as length of stay after lung resection.

Prediction-model studies require evaluation beyond discrimination alone. Calibration, overall prediction error, clinical net benefit, internal validation, temporal validation, and external validation are needed to determine whether estimated risks remain reliable in new patients [6,13,14,15,16,17,18]. Cross-country validation is especially demanding because variable definitions, monitoring density, procedure spectrum, discharge practice, and outcome recording may differ even when nominally similar predictors are available [6].

We therefore developed a baseline perioperative model and a BP-enhanced model for PLOS after wedge resection, segmentectomy, or lobectomy. We used a Chinese hospital cohort for development and later temporal validation, followed by direct validation of the unchanged models in a procedure-restricted Korean VitalDB cohort. The primary aims were to distinguish local temporal performance from geographic transportability and to test whether four compact BP summaries added reproducible predictive information. These aims concern prediction, not the causal effect of BP on length of stay.

## 2. Materials and Methods

### 2.1. Study Design and Reporting

This retrospective cohort study comprised prediction-model development, nested internal validation, local temporal validation, and geographic external validation. Reporting was structured according to the updated Transparent Reporting of a Multivariable Prediction Model for Individual Prognosis or Diagnosis plus Artificial Intelligence (TRIPOD + AI) guidance [16]. Methodological decisions were also informed by PROBAST domains with regard to participants, predictors, outcomes, and analysis [14].

### 2.2. Local Cohort

The local data source contained 720 rows representing 720 unique adult patients who underwent one eligible lung resection at Shanghai Chest Hospital between 2021 and 2025. Eligible procedures were wedge resection, segmentectomy, and lobectomy. Procedure codes were harmonized as 0 = wedge resection, 1 = segmentectomy, and 2 = lobectomy. Patients treated from 2021 through 2024 were assigned to model development, whereas patients treated during 2025 were reserved as a non-random temporal-validation cohort. The original clinical source file was not modified; all harmonization and modeling used reproducible derived datasets.

### 2.3. VitalDB External Cohort

VitalDB is an open perioperative database containing high-resolution intraoperative biosignals and clinical information from Seoul National University Hospital, Republic of Korea [12]. The available thoracic source cohort contained 1111 cases; it was not used in full for external validation. To align the operative population with the local cohort, we retained only exact procedure names corresponding to lung wedge resection (*n* = 231), segmentectomy (*n* = 63), or lobectomy (*n* = 332). This excluded 485 other thoracic procedures and produced a 626-patient external-validation cohort. A case was considered BP-eligible when track processing produced all four BP predictors used by the BP-enhanced model. This criterion was met by 624 patients. Of the two excluded from paired comparison, one had no ART_MBP file and one had values outside the prespecified physiologic range; both had PLOS = 0 (postoperative stays, 2.44 and 2.58 days). Baseline-model performance was evaluated in all 626 patients and separately in the 624-patient paired sample.

### 2.4. Outcome Definition

The primary outcome was PLOS, defined before model fitting as postoperative length of stay > 4 days. This cutoff was an operational resource-use definition chosen for consistent model development, rather than a validated clinical state or a specific complication. For the local cohort, postoperative length of stay was available directly in days, but the source data dictionary did not document the exact time origin or rounding convention. Twenty-four local records had an original binary PLOS flag of 0 despite a continuous postoperative stay of 5–7 days. The analysis therefore applied the prespecified rule to the continuous field for all local patients, yielding 249 events among 536 development patients and 74 among 184 temporal-validation patients. The discrepancy was retained in the data-quality audit and could not be attributed to a specific cause from the available records. In VitalDB, postoperative length of stay was calculated as hospital discharge time minus operation end time and converted from seconds to days. Non-positive external stays were treated as invalid. Nominally identical thresholds may not represent the same clinical state or discharge process across centers.

### 2.5. Prespecified Predictors

Predictors were specified before final model fitting based on clinical relevance, availability by the end of surgery, and availability under a common variable name in both cohorts. No univariable screening or validation-cohort-driven predictor selection was performed. The baseline model included age, sex, body mass index, American Society of Anesthesiologists (ASA) physical status, diabetes mellitus, hypertension, preoperative hemoglobin, albumin, and creatinine, procedure type, surgical approach, surgical duration, and estimated blood loss. Hemoglobin and albumin were converted from g/L to g/dL in the local cohort, and creatinine was converted from micromoles/L to mg/dL. Surgical duration and estimated blood loss were transformed using log(1 + *x*). Detailed source fields, units, coding, and known differences are provided in Table S6.

The BP-enhanced model added four prespecified features: mean MAP, log-transformed time-weighted average (TWA) MAP deficit below 65 mmHg, the fraction of the source-defined observation duration with MAP below 65 mmHg, and the log-transformed duration of the longest MAP-below-65 episode. The source-specific denominators are stated below because they were not identical across cohorts. Minimum MAP was not included because it is sensitive to single artifacts and minor rounding inconsistencies. Because surgical duration and blood loss were known only near the end of the procedures, the intended prediction time was the end of surgery. The models were evaluated as risk-stratification tools, not as causal estimators. Potential uses such as postoperative recovery review or discharge-resource planning remain proposed applications and were not tested as interventions.

### 2.6. BP Processing and Harmonization

All local patients underwent arterial catheterization. The anesthesia information system stored invasive MAP values every 5 min from anesthesia start to anesthesia end. Anesthesiologists reviewed the intraoperative anesthesia record and corrected artifactual values at the corresponding time points before export; no additional numeric artifact filter was applied by the research script. Missing scheduled MAP entries were completed by the anesthesiologist before calculation, and the exported series contained no missing MAP values. The research team then derived the four summaries in Python 3.12. If MAP_i denotes the ith valid 5 min MAP value and T_valid denotes valid monitored time, calculated from the anesthesia-start and anesthesia-end timestamps, mean MAP is the arithmetic mean of all MAP_i values; TWA deficit below 65 mmHg is sum [max (65 − MAP_i, 0) × 5]/T_valid; the low-MAP fraction is sum [I (MAP_i < 65) × 5]/T_valid. The longest episode was the largest number of consecutive MAP_i values below 65 mmHg multiplied by 5 min. A value of 65 mmHg or higher terminated an episode. Supplementary Code S1 implements these calculations.

For the primary VitalDB derivation, invasive ART_MBP tracks were sorted by time and values outside 20–200 mmHg were removed. Successive time intervals were truncated to 0–30 s for exposure integration; the final interval was filled with the within-track median positive interval of 30 s or less. A 2 s default was prespecified if no such median could be obtained, although it was not required in any eligible case. MAP deficit below 65 mmHg was integrated over valid monitored time to obtain TWA. Consecutive values below 65 mmHg were summarized into episodes, and low-MAP minutes were divided by surgical duration for the fraction measure. Among 3,746,159 raw values in the 626 procedure-restricted cases, 305,456 (8.15%) were removed as outside 20–200 mmHg. The 30 s interval cap affected 898 intervals in 417 cases; 11,134 non-positive intervals were assigned zero duration. One track was unavailable and one contained no value within 20–200 mmHg, leaving 624 BP-eligible patients (Table S7).

The primary track summaries used the complete available ART_MBP files; 75.4% of retained values fell within the recorded operation-start to operation-end window. We therefore performed a processing sensitivity analysis that explicitly restricted VitalDB MAP to this window and represented each fixed 1-, 2-, or 5-min bin by its median. The four BP predictors were recalculated from these equally spaced values. These analyses assess sensitivity to the external processing window and sampling interval; they do not establish equivalence with the unaudited local summaries.

### 2.7. Missing Data

Missing values were handled within each model-fitting pipeline to prevent information leakage. Numeric predictors were imputed using the training-fold median, with an additional missingness indicator. Categorical predictors were imputed using the most frequent training-fold category and one-hot encoded. The same fitted preprocessing transformation was then applied without modification to the corresponding validation data. Predictor missingness by cohort is reported in Table S1, and all development-set imputation and scaling parameters are reported in Table S8. Sensitivity analyses evaluated the unchanged submitted models in complete cases and refitted both models using iterative numeric imputation without missingness indicators; categorical processing was otherwise unchanged.

### 2.8. Model Development and Validation

The primary algorithm was L2-penalized logistic regression. Penalized regression was selected for transparency and coefficient shrinkage in the available sample [15,19,20]. The regularization parameter C was selected independently for each model from 0.01, 0.03, 0.1, 0.3, 1, 3, and 10 by inner cross-validation optimizing negative log loss. The random seed was 20260715.

Internal performance was estimated using nested cross-validation in the 2021–2024 development cohort. For PLOS, the outer loop used 10 stratified folds and each training portion used five-fold inner cross-validation. After hyperparameter selection, the model was refitted to the complete development cohort and applied once to the untouched 2025 temporal-validation cohort and the Korean external cohort. Neither validation cohort was used for predictor selection, regularization selection, or model updating.

For both final PLOS models, nested cross-validation selected C = 0.10 for refitting on the complete development cohort. The preprocessing order was numeric imputation with missingness indicators followed by robust scaling, and categorical most-frequent imputation followed by one-hot encoding. OneHotEncoder retained every observed category (drop = None) and ignored previously unseen validation categories. The logistic model included an intercept; with the liblinear solver and intercept_scaling = 1, the intercept was represented by a synthetic feature and was subject to regularization. Consequently, individual coefficients depend on this full-level penalized coding and should not be interpreted as conventional reference-category effects. Complete coefficients are reported in Table S2, fold-specific regularization selections in Table S3, and preprocessing parameters in Table S8. Frozen pipelines were serialized to permit exact regeneration of predicted probabilities.

The development cohort contained 249 events among 536 patients. The baseline and BP-enhanced pipelines contained 26 and 31 transformed slope terms, respectively. Because no prospective sample-size calculation had been performed, we added a post hoc context assessment using the Riley framework [17,20], with temporal-validation AUROC and development event prevalence as inputs. This assessment evaluates potential overfitting and precision limitations; it is not presented as an a priori design calculation (Supplementary Methods and Table S9).

### 2.9. Performance Measures

Discrimination was quantified using AUROC and average precision. Calibration was summarized by calibration intercept, calibration slope, and flexible logistic-spline calibration curves. Pointwise uncertainty bands were estimated by bootstrap resampling, and predicted-risk distributions were displayed by outcome. Overall prediction error was measured using the Brier score. Clinical utility was explored using decision-curve analysis over threshold probabilities of 0.05–0.70, with bootstrap uncertainty bands and treat-all and treat-none comparators [18,21]. Because no validated action threshold or intervention was prespecified, decision curves were considered descriptive rather than evidence of clinical benefit.

Ninety-five percent confidence intervals were estimated using 1000 bootstrap resamples. Incremental BP value was quantified by paired bootstrap differences in AUROC, average precision, Brier score, calibration intercept, and calibration slope, with the BP-enhanced model minus the baseline model as the contrast. A positive Brier-score difference indicates worse prediction error.

### 2.10. Model Interpretation

Exploratory linear SHAP analyses are described in the Supplementary Methods and Supplementary Figure S2. These values describe contributions to the fitted model on the log-odds scale. They do not estimate causal effects, independent predictor effects, or reasons for transportability failure.

### 2.11. Software

Analyses were performed in Python 3.12 using pandas 2.2.3, NumPy 2.3.5, SciPy 1.16.3, and scikit-learn 1.9.0. All preprocessing, model fitting, hyperparameter selection, and validation steps were implemented in reproducible scripts. The analysis code did not alter either raw source dataset.

## 3. Results

### 3.1. Cohort Characteristics

**Figure 1:**
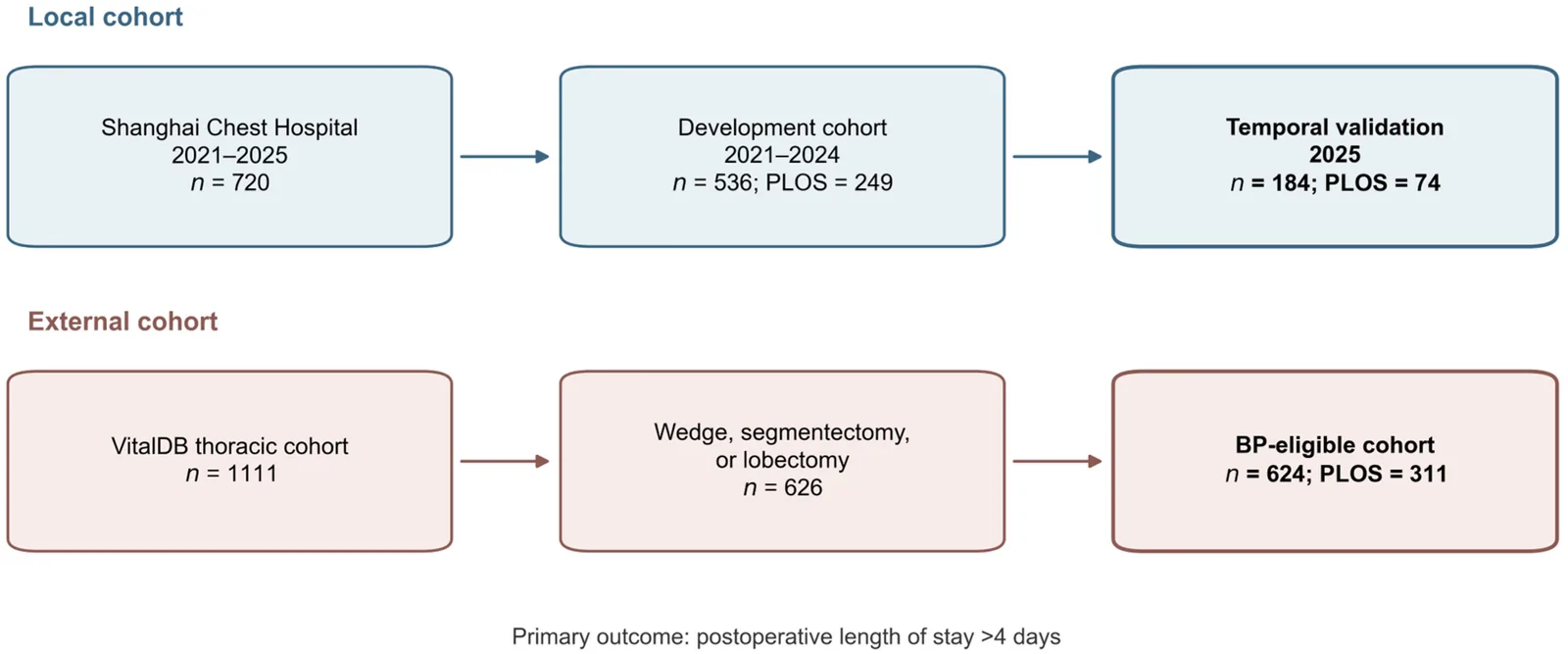
Cohort construction and validation design. The local cohort was divided chronologically into development and temporal-validation samples. The procedure-restricted VitalDB cohort provided geographic external validation. Blue indicates the local cohorts and brown indicates the external cohort. PLOS, prolonged postoperative length of stay; BP, blood pressure.

The local cohort included 536 development patients and 184 temporal-validation patients; PLOS occurred in 249 (46.5%) and 74 (40.2%), respectively. The procedure-restricted VitalDB cohort included 626 patients, of whom 311 (49.7%) experienced PLOS. Figure 1 summarizes the cohort construction.

The cohorts differed in several clinically important respects (Table 1). Median age was 60 years in local development, 58 years in local temporal validation, and 64 years in VitalDB. Lobectomy accounted for 13.4%, 14.7%, and 53.0% of cases, respectively, whereas segmentectomy accounted for 40.5%, 38.6%, and 10.1%. VATS was more frequent in VitalDB (95.5%) than locally (76.7–80.4%). The distribution of BP burden also differed; median TWA MAP deficit below 65 mmHg was 0.00 mmHg in both local cohorts and 0.32 mmHg in VitalDB. Absolute standardized mean differences between local temporal validation and development were below 0.14 for all assessed predictors. In contrast, external-versus-development SMDs were 0.974 for mean MAP, 0.900 for TWA MAP deficit, 0.694 for the low-MAP fraction, 0.927 for lobectomy, −0.747 for segmentectomy, and 0.566 for VATS, with additional imbalances in age, laboratory values, surgical duration, and blood loss (Table S6; Figure S1). These results demonstrated incomplete predictor overlap despite procedure-family restriction.

### 3.2. Baseline Model Performance

Both final PLOS models used C = 0.10. Across the 10 outer folds, the selected C values were 0.10 or 0.30, indicating that the regularization choice remained within a narrow range (Table S3). Complete fitted coefficients are provided in Table S2.

The baseline model achieved a nested internal AUROC of 0.714 (95% CI, 0.667–0.756) and average precision of 0.636 (0.571–0.704). Its calibration intercept was 0.001 and calibration slope was 0.925, with a Brier score of 0.212 (0.197–0.227).

During local 2025 temporal validation, discrimination was similar to nested internal performance: AUROC 0.719 (95% CI, 0.640–0.788) and average precision 0.652 (0.556–0.756). The calibration intercept was −0.246 (95% CI, −0.548 to 0.050), the calibration slope was 1.113 (0.703–1.600), and the Brier score was 0.209 (0.188–0.232).

In all 626 Korean patients, the baseline model had an AUROC of 0.624 (95% CI, 0.577–0.671), average precision of 0.624 (0.571–0.683), Brier score of 0.245 (0.232–0.259), calibration intercept of −0.123 (−0.297 to 0.047), and calibration slope of 0.573 (0.363–0.798). Results were nearly identical in the 624-patient BP-eligible sample: AUROC 0.623 (0.579–0.666), average precision 0.625 (0.572–0.685), Brier score 0.245 (0.232–0.259), calibration intercept −0.117 (−0.290 to 0.056), and calibration slope 0.570 (0.356–0.795). The unchanged baseline model therefore had limited external discrimination and substantially attenuated calibration; it was not suitable for direct cross-center implementation.

### 3.3. Incremental Value of BP Features

The BP-enhanced model achieved a nested internal AUROC of 0.721 (95% CI, 0.674–0.762), average precision of 0.657 (0.590–0.726), and Brier score of 0.211 (0.196–0.228). Relative to the baseline model, BP features changed internal AUROC by +0.007 (95% CI, −0.004 to +0.018), average precision by +0.021 (+0.001 to +0.040), and Brier score by −0.001 (−0.004 to +0.003).

In temporal validation, the BP-enhanced model produced an AUROC of 0.725 (95% CI, 0.649–0.793), average precision of 0.659 (0.561–0.756), Brier score of 0.206 (0.184–0.229), calibration intercept of −0.240 (−0.536 to 0.063), and calibration slope of 1.112 (0.719–1.579). The paired temporal increments were +0.006 (95% CI, −0.009 to +0.021) for AUROC, +0.007 (−0.019 to +0.032) for average precision, −0.002 (−0.006 to +0.002) for Brier score, +0.005 (−0.018 to +0.026) for calibration intercept, and −0.001 (−0.092 to +0.085) for calibration slope. The confidence intervals did not establish a reproducible temporal improvement.

In the Korean external validation, the BP-enhanced model achieved an AUROC of 0.615 (95% CI, 0.572–0.661), average precision of 0.613 (0.559–0.676), Brier score of 0.249 (0.234–0.263), calibration intercept of −0.225 (−0.402 to −0.058), and calibration slope of 0.534 (0.331–0.752). BP features changed external AUROC by −0.007 (95% CI, −0.022 to +0.008), average precision by −0.012 (−0.031 to +0.007), Brier score by +0.003 (−0.001 to +0.008), calibration intercept by −0.108 (−0.127 to −0.090), and calibration slope by −0.036 (−0.105 to +0.027). The external data did not support an increment in BP value.

In the operation-window processing sensitivity analysis, 5 min binning increased the proportion of VitalDB patients with zero TWA MAP deficit to 71.2%, similar to 67.9% in the local development cohort. Nevertheless, external discrimination did not improve. Among 622 patients evaluable under every binning scenario, BP-enhanced minus baseline differences across 1, 2, and 5 min binning were −0.008 to −0.007 for AUROC, −0.016 to −0.014 for average precision, and +0.0049 to +0.0050 for Brier score. Calibration-slope differences were −0.030 to −0.027, with all 95% CIs crossing zero (Table S5).

Missing-data sensitivity analyses gave the same substantive conclusion (Table S10). In 356 complete cases (200 events), baseline and BP-enhanced AUROCs were 0.602 and 0.605, respectively, with Brier scores of 0.246 and 0.245. After iterative numeric imputation without missingness indicators in the 624-patient paired sample, AUROCs were 0.622 and 0.613, and Brier scores were 0.245 and 0.249. Neither strategy showed a consistent BP increment. Model performance is summarized in Table 2, and paired incremental estimates are summarized in Table 3.

**Figure 2:**
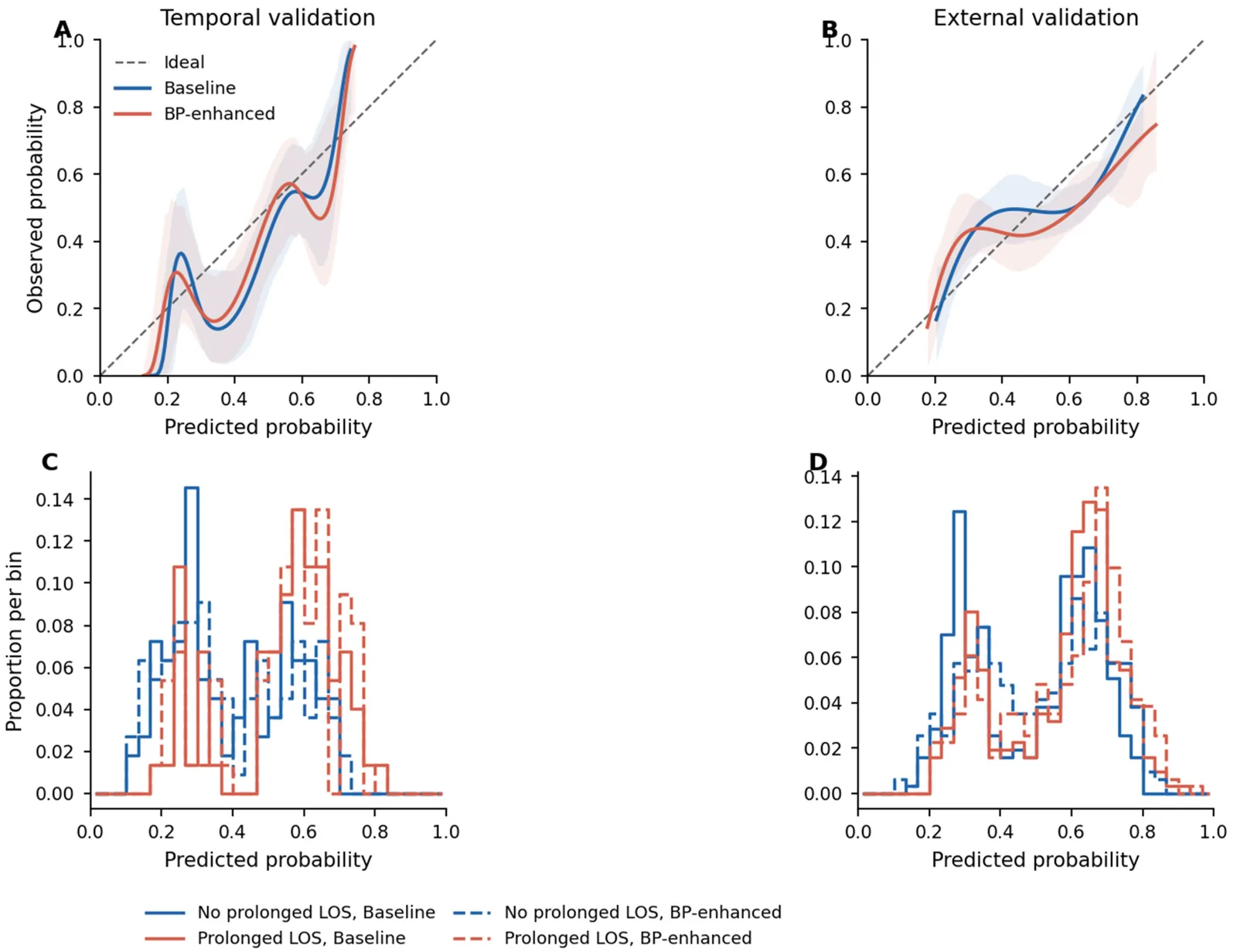
Flexible calibration and predicted-risk distributions during local temporal and Korean external validation. (**A**,**B**) Logistic-spline calibration curves; shaded regions are pointwise bootstrap 95% intervals, with blue denoting the baseline model and red denoting the BP-enhanced model; the diagonal denotes ideal calibration. (**C**,**D**) Predicted-risk distributions by observed outcome; solid lines denote the baseline model and dashed lines the BP-enhanced model. The local temporal cohort included 184 patients with 74 events. Paired external analyses included 624 patients with 311 events. BP, blood pressure; LOS, length of stay.

**Figure 3:**
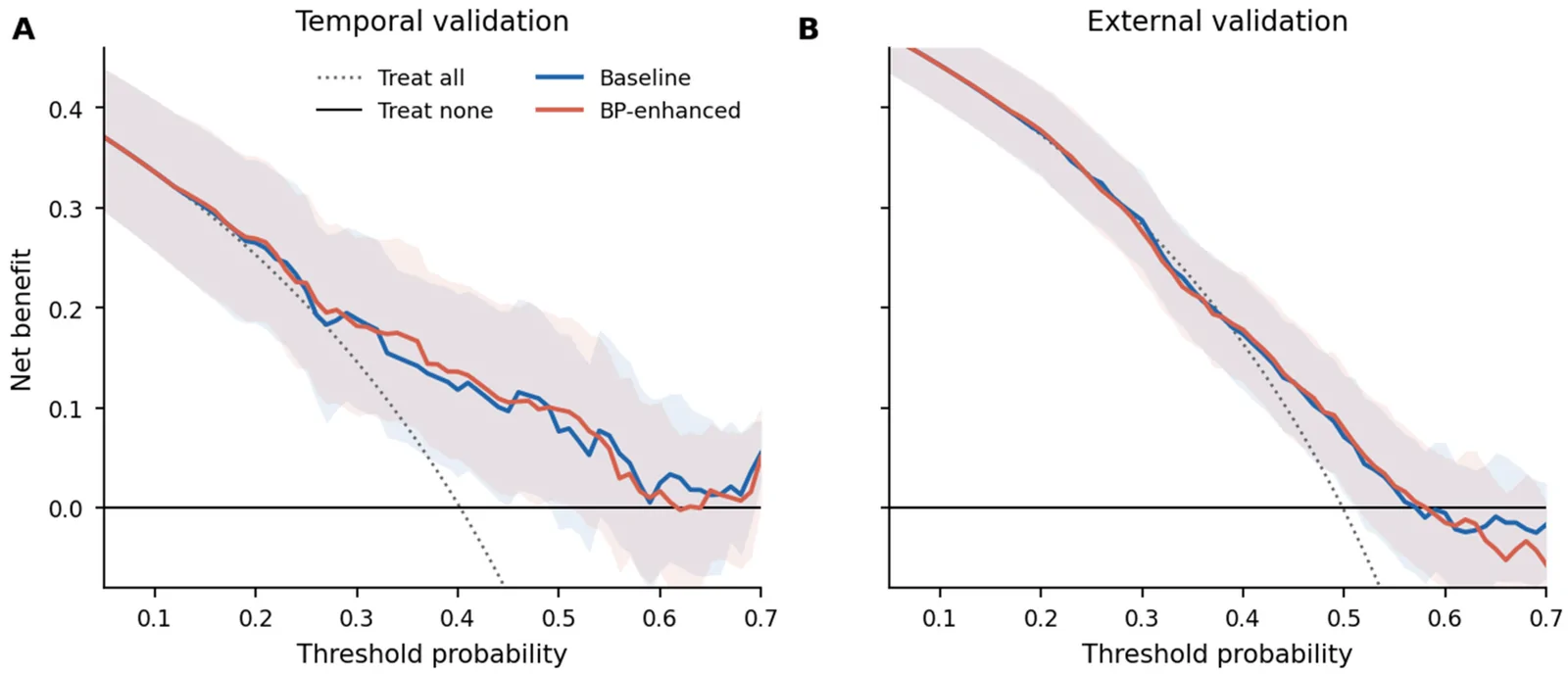
Exploratory decision-curve analysis during (**A**) local temporal validation and (**B**) Korean external validation. Shaded regions are pointwise bootstrap 95% intervals, with blue denoting the baseline model and red denoting the BP-enhanced model. Treat-all and treat-none strategies are shown for comparison. Threshold probabilities range from 0.05 to 0.70; no threshold was prespecified for clinical action. BP, blood pressure.

Discrimination and calibration results for local temporal and Korean external validation are summarized in Figure 2. Paired incremental comparisons across internal, temporal, and external validation are summarized in Figure 3.

### 3.4. Calibration and Decision-Curve Analysis

Temporal calibration estimates were compatible with a slope of one, but their intervals were wide because only 74 events were available. External calibration slopes were substantially below one, and the flexible curves showed compressed risk separation (Figure 2). These patterns are consistent with overfitted predictor effects, altered predictor-outcome relations, or both; the present data cannot distinguish among these explanations.

Both models had positive estimated net benefit relative to treat-none over portions of the evaluated threshold range, but uncertainty bands were wide and treat-all remained competitive at lower thresholds. The BP-enhanced curve did not consistently separate from the baseline curve in either validation cohort (Figure 3). Because the study did not prespecify an action linked to a validated threshold, these findings are descriptive and do not establish clinical utility.

### 3.5. Sample-Size Context and Supplementary Analyses

The development sample provided 9.58 and 8.03 events per transformed slope term for the baseline and BP-enhanced models, respectively. In the post hoc Riley-framework assessment, the corresponding suggested development sizes were 1484 patients (690 events) and 1673 patients (778 events), exceeding the available 536 patients and 249 events (Table S9). Penalization and nested validation reduced but did not remove the resulting overfitting risk. For external validation, 624 patients met the prespecified precision scenarios for AUROC and observed-to-expected ratio but not for a calibration-slope 95% CI width of 0.20.

BP burden features were strongly correlated. In development, Spearman correlations were 0.811 for TWA deficit versus low-MAP fraction, 0.804 for TWA deficit versus longest episode, and 0.955 for low-MAP fraction versus longest episode. In 300 bootstrap refits, most BP coefficient intervals crossed zero, supporting redundancy and coefficient instability rather than independently interpretable BP effects (Table S11). Descriptive SHAP analyses were moved to the Supplementary Materials and were not used for causal or independent-effect inference.

## 4. Discussion

### 4.1. Principal Findings

This study yielded three principal findings. First, baseline-model discrimination in the later local cohort was similar to nested internal performance, although calibration estimates remained imprecise. Second, direct application of the unchanged models to VitalDB produced limited discrimination and calibration slopes near 0.5, precluding direct cross-center use. Third, the four BP summaries did not provide reproducible incremental value in temporal validation, external validation, complete-case analysis, alternative imputation, or operation-window sampling analyses. The isolated internal average-precision increment should therefore be interpreted as a development-sample signal that did not replicate.

These findings separate local temporal performance from geographic transportability. They also distinguish predictive contribution from causality. Neither penalized coefficients nor SHAP values show that a BP exposure causes prolonged stay, and the absence of incremental prediction does not establish that intraoperative BP is clinically unimportant for other outcomes.

### 4.2. Comparison with Previous Studies

Previous thoracic studies have developed risk models for prolonged hospitalization using comorbidities, pulmonary function, procedure characteristics, operative duration, laboratory variables, and postoperative complications [3,4,5]. A recent machine-learning study of 6767 VATS patients similarly identified surgical duration, age, creatinine, and hemoglobin among important PLOS predictors [5]. Enhanced-recovery studies further show that postoperative LOS is responsive to differences in multidisciplinary pathways and perioperative practice [2]. Our baseline model used a compact set of corresponding variables and achieved comparable moderate temporal discrimination, while adding direct geographic validation, calibration, and net-benefit assessment rather than relying on apparent discrimination alone.

Large perioperative cohorts have reported associations between the depth and duration of intraoperative hypotension and myocardial injury, acute kidney injury, cardiovascular events, or mortality [7,8,9,10]. Those associations provide a rationale for evaluating BP burden as a predictor, but they do not guarantee added value for a recovery- and process-sensitive outcome such as PLOS. In the present data, strong correlations among BP summaries and unstable bootstrap coefficients further reduced the likelihood that four related features would add robust information beyond procedure and perioperative variables.

### 4.3. External Transportability and Clinical Implications

The external decline was accompanied by substantial case-mix and measurement shift. Several external-versus-development SMDs exceeded 0.5, including procedure mix, surgical approach, and three BP burden summaries. These differences are consistent with altered model applicability, but the current study cannot determine how much of the performance loss was caused by each source of shift, nor can it exclude model misspecification. The result should therefore be understood as limited unchanged transportability in this setting, not as proof of a national effect or of one causal mechanism.

Outcome transportability is also uncertain. The >4-day cutoff was prespecified before modeling and applied to a continuous LOS field, but it is an operational resource-use threshold rather than a defined clinical state. The local data dictionary did not document the exact time origin or rounding convention, whereas VitalDB LOS was measured from operation end to hospital discharge. Discharge criteria, chest-tube practice, enhanced-recovery pathways, social support, and bed-management processes may further change the meaning of the same numerical threshold across hospitals.

BP processing was not equivalent across sources despite invasive measurement in both cohorts. Local summaries used anesthesiologist-reviewed 5 min MAP records over the anesthesia interval, with valid monitored time calculated from the anesthesia-start and anesthesia-end timestamps as the denominator for both TWA deficit and low-MAP fraction. VitalDB summaries were reconstructed from high-frequency tracks after numeric artifact filtering and interval truncation; TWA likewise used valid monitored time, whereas the low-MAP fraction used surgical duration. The primary VitalDB summary also included track periods outside the recorded operation window, although 1-, 2-, and 5-min sensitivity analyses gave the same no-increment conclusion. These analyses show that the conclusion was not reversed by the tested external processing rules; they do not prove that the local and VitalDB BP measurements are interchangeable.

The simpler baseline model is the more defensible starting point for future evaluation because routine extraction of the four BP summaries added complexity without reproducible predictive gain. This preference does not make the baseline model ready for practice; its external calibration was inadequate, no clinical action threshold was validated, and net-benefit analyses were exploratory. If evaluated prospectively at the end of surgery, a risk estimate might prompt structured recovery review, discharge-resource planning, or closer reassessment rather than automatic treatment. Such a workflow would require predefined actions, center-specific calibration, and prospective impact evaluation before deployment.

### 4.4. Strengths and Limitations

This study has several strengths. It used a later-year temporal-validation cohort rather than a random split, applied nested cross-validation and penalization during development, evaluated discrimination, calibration, prediction error, and exploratory net benefit, quantified BP increments with paired bootstrap intervals, and performed direct external validation without model updating. The revision also reports flexible calibration with uncertainty, predicted-risk distributions, predictor SMDs, missing-data sensitivity analyses, BP-feature correlations, coefficient stability, and full 626-patient baseline performance.

This study also has important limitations. First, the retrospective single-center development sample was smaller than suggested by the post hoc Riley-framework assessment. L2 penalization and nested validation reduce but do not eliminate overfitting risk. The temporal cohort contained only 74 events, and the external sample did not meet the prespecified precision target for calibration slope. Although BP was measured invasively in both cohorts and the local calculation is described in detail, sampling resolution, artifact handling, observation windows, and the denominators of the duration-based summaries differed from VitalDB; computational equivalence was therefore not established. Moreover, the local LOS time origin and rounding were not documented, 24 binary source flags disagreed with the continuous-field definition, and a >4-day threshold may have different clinical meaning across discharge systems. Missing laboratory and blood-loss values were more frequent externally; sensitivity analyses did not reverse the findings but cannot exclude informative missingness. Furthermore, the harmonized set omitted pulmonary-function and postoperative-complication variables; the latter would occur after the intended end-of-surgery prediction time. Finally, no threshold-linked intervention or prospective workflow was evaluated.

## 5. Conclusions

The baseline perioperative model showed similar discrimination in a later local cohort but limited unchanged transportability to VitalDB. Four intraoperative BP summaries did not add reproducible predictive value across temporal, external, and sensitivity analyses. Current evidence therefore favors the simpler baseline specification for further study, not direct implementation. Prospective multicenter work should harmonize BP acquisition, prediction time, and LOS definitions, then evaluate model updating, calibration, actionable thresholds, and clinical impact.

## Figures and Tables

**Table 1:** Cohort characteristics.

Characteristic	Local Development 2021–2024 (*n* = 536)	Local Temporal 2025 (*n* = 184)	VitalDB Korea External (*n* = 626)	Missing Dev, *n* (%)	Missing Temporal, *n* (%)	Missing External, *n* (%)	SMD Temporal vs. Dev	SMD External vs. Dev
Age, years	60.0 [52.0–68.0]	58.0 [53.0–68.5]	64.0 [56.2–71.0]	0 (0.0%)	0 (0.0%)	0 (0.0%)	−0.008	0.224
Male sex	289 (53.9%)	107 (58.2%)	329 (52.6%)	0 (0.0%)	0 (0.0%)	0 (0.0%)	0.085	−0.027
Body mass index, kg/m^2^	23.7 [21.2–25.6]	23.2 [21.1–25.7]	23.3 [21.2–25.4]	0 (0.0%)	0 (0.0%)	0 (0.0%)	−0.058	−0.058
ASA physical status III–IV	50 (9.3%)	21 (11.4%)	62 (9.9%)	0 (0.0%)	0 (0.0%)	15 (2.4%)	See Table S6	See Table S6
Diabetes mellitus	55 (10.3%)	17 (9.2%)	77 (12.3%)	0 (0.0%)	0 (0.0%)	0 (0.0%)	−0.034	0.064
Hypertension	137 (25.6%)	41 (22.3%)	213 (34.0%)	0 (0.0%)	0 (0.0%)	0 (0.0%)	−0.077	0.186
Hemoglobin, g/dL	13.7 [12.9–14.5]	13.5 [12.9–14.3]	13.1 [12.2–14.2]	24 (4.5%)	3 (1.6%)	70 (11.2%)	−0.097	−0.422
Albumin, g/dL	4.27 [4.08–4.42]	4.29 [4.04–4.44]	4.20 [4.00–4.40]	24 (4.5%)	3 (1.6%)	84 (13.4%)	−0.063	−0.306
Creatinine, mg/dL	0.76 [0.65–0.88]	0.72 [0.64–0.87]	0.80 [0.68–0.95]	24 (4.5%)	3 (1.6%)	83 (13.3%)	−0.092	0.299
Wedge resection	247 (46.1%)	86 (46.7%)	231 (36.9%)	0 (0.0%)	0 (0.0%)	0 (0.0%)	0.013	−0.187
Segmentectomy	217 (40.5%)	71 (38.6%)	63 (10.1%)	0 (0.0%)	0 (0.0%)	0 (0.0%)	−0.039	−0.747
Lobectomy	72 (13.4%)	27 (14.7%)	332 (53.0%)	0 (0.0%)	0 (0.0%)	0 (0.0%)	0.036	0.927
VATS approach	411 (76.7%)	148 (80.4%)	598 (95.5%)	0 (0.0%)	0 (0.0%)	0 (0.0%)	0.092	0.566
Surgical duration, min	120 [93–153]	120 [95–145]	125 [100–161]	0 (0.0%)	0 (0.0%)	0 (0.0%)	−0.015	0.280
Estimated blood loss, mL	100 [50–168]	100 [50–140]	100 [50–200]	0 (0.0%)	0 (0.0%)	210 (33.5%)	−0.078	0.224
Mean intraoperative MAP, mmHg	80.2 [75.6–82.8]	79.6 [77.1–82.3]	85.1 [80.0–90.7]	9 (1.7%)	3 (1.6%)	2 (0.3%)	0.067	0.974
TWA MAP < 65 mmHg, mmHg	0.00 [0.00–0.01]	0.00 [0.00–0.01]	0.32 [0.13–0.57]	0 (0.0%)	0 (0.0%)	2 (0.3%)	0.042	0.900
Low-MAP fraction, source-defined denominator	0.000 [0.000–0.000]	0.000 [0.000–0.000]	0.041 [0.013–0.108]	0 (0.0%)	0 (0.0%)	2 (0.3%)	−0.044	0.694
Longest MAP < 65 episode, min	0.0 [0.0–0.0]	0.0 [0.0–0.0]	2.2 [0.7–4.7]	0 (0.0%)	0 (0.0%)	2 (0.3%)	−0.107	0.448
Postoperative LOS, days	4.0 [3.0–5.0]	4.0 [4.0–5.0]	3.6 [2.5–5.5]	Not applicable	Not applicable	Not applicable	Not applicable	Not applicable
Prolonged postoperative LOS	249 (46.5%)	74 (40.2%)	311 (49.7%)	Not applicable	Not applicable	Not applicable	Not applicable	Not applicable
ICU use	68 (12.7%)	18 (9.8%)	379 (60.5%)	Not applicable	Not applicable	Not applicable	Not applicable	Not applicable

Values are median [interquartile range] or *n* (%). ASA, American Society of Anesthesiologists; BP, blood pressure; ICU, intensive care unit; MAP, mean arterial pressure; PLOS, prolonged postoperative length of stay; TWA, time-weighted average; VATS, video-assisted thoracoscopic surgery.

**Table 2:** Performance of the prolonged postoperative length-of-stay models.

Validation	Model	*n*/Events	AUROC (95% CI)	Average Precision (95% CI)	Brier Score (95% CI)	Calibration Intercept (95% CI)	Calibration Slope (95% CI)
Local temporal validation (2025)	Baseline model	184/74	0.719 (0.640 to 0.788)	0.652 (0.556 to 0.756)	0.209 (0.188 to 0.232)	−0.246 (−0.548 to 0.050)	1.113 (0.703 to 1.600)
Local temporal validation (2025)	BP-enhanced model	184/74	0.725 (0.649 to 0.793)	0.659 (0.561 to 0.756)	0.206 (0.184 to 0.229)	−0.240 (−0.536 to 0.063)	1.112 (0.719 to 1.579)
VitalDB external validation (paired sample)	Baseline model	624/311	0.623 (0.579 to 0.666)	0.625 (0.572 to 0.685)	0.245 (0.232 to 0.259)	−0.117 (−0.290 to 0.056)	0.570 (0.356 to 0.795)
VitalDB external validation (paired sample)	BP-enhanced model	624/311	0.615 (0.572 to 0.661)	0.613 (0.559 to 0.676)	0.249 (0.234 to 0.263)	−0.225 (−0.402 to −0.058)	0.534 (0.331 to 0.752)
VitalDB external validation (all patients)	Baseline model	626/311	0.624 (0.577 to 0.671)	0.624 (0.571 to 0.683)	0.245 (0.232 to 0.259)	−0.123 (−0.297 to 0.047)	0.573 (0.363 to 0.798)

AUROC, area under the receiver operating characteristic curve; BP, blood pressure; CI, confidence interval; PLOS, prolonged postoperative length of stay.

**Table 3:** Incremental value of BP features relative to the baseline model.

Validation	Delta AUROC (95% CI)	Delta Average Precision (95% CI)	Delta Brier Score (95% CI)	Delta Calibration Intercept (95% CI)	Delta Calibration Slope (95% CI)
Local temporal validation (2025)	0.006 (−0.009 to 0.021)	0.007 (−0.019 to 0.032)	−0.002 (−0.006 to 0.002)	0.005 (−0.018 to 0.026)	−0.001 (−0.092 to 0.085)
VitalDB external validation (paired sample)	−0.007 (−0.022 to 0.008)	−0.012 (−0.031 to 0.007)	0.003 (−0.001 to 0.008)	−0.108 (−0.127 to −0.090)	−0.036 (−0.105 to 0.027)

Positive differences favor the BP-enhanced model except for Brier score, for which negative differences indicate improvement. AUROC, area under the receiver operating characteristic curve; BP, blood pressure; CI, confidence interval.

## Data Availability

The local clinical data contain protected patient information and are not publicly available. De-identified data may be made available by the corresponding author upon reasonable request and subject to institutional and ethical approval. VitalDB is publicly accessible at https://vitaldb.net (accessed on 15 April 2026). The analysis code and non-identifiable aggregate results are available from the corresponding author upon reasonable request.

## Supplementary Materials

The following supporting information can be downloaded at https://www.mdpi.com/article/10.3390/jcm15176887/s1, Code S1: Local invasive-BP summary generation; Table S1: Predictor missingness; Table S2: Final model coefficients; Table S3: Penalization selection; Table S4: Exploratory ICU performance; Table S5: Operation-window fixed-interval VitalDB sensitivity; Table S6: Predictor standardized mean differences; Table S7: VitalDB ART_MBP quality-control audit; Table S8: Development-set preprocessing parameters; Table S9: Post hoc sample-size context; Table S10: Missing-data sensitivity analyses; Table S11A: BP-feature Spearman correlations; Table S11B: Bootstrap coefficient stability; Figure S1: Predictor shift and overlap. (A), Standardized mean differences comparing local temporal validation and VitalDB external validation with local development; the shaded region denotes absolute SMD < 0.10. (B–E), Empirical cumulative distributions of the four BP summaries. Positive and negative SMD signs indicate direction only; magnitude reflects imbalance. BP, blood pressure; MAP, mean arterial pressure; TWA, time-weighted average; VATS, video-assisted thoracoscopic surgery; Figure S2: Descriptive model contributions for PLOS. (a,b), Mean absolute linear SHAP values in local development for the baseline and BP-enhanced models. (c), Patient-level contribution distributions for selected numeric predictors. (d), Contributions from the unchanged BP-enhanced model in local development and VitalDB. Values are on the fitted model log-odds scale and are descriptive; they do not represent causal effects, independent predictor effects, or explanations for transportability failure. SHAP, Shapley additive explanations; BP, blood pressure; MAP, mean arterial pressure; TWA, time-weighted average.
