# Supplementary material for: Prolonged Length of Stay After Lung Resection: A Prediction Model That Loses Accuracy Across Hospitals, with No Added Benefit from Blood Pressure Data
Source: J Clin Med. 2026 Sep 5;15(17):6887. doi: 10.3390/jcm15176887 (PMC13566256; doi:10.3390/jcm15176887)
Supplement: Supplementary file 1 [file jcm-15-06887-s001.zip › Supplementary Materials.pdf]

Supplementary Materials

Article: Prolonged Length of Stay After Lung Resection: A Prediction Model That Loses Accuracy Across Hospitals, with No Added Benefit from Blood Pressure Data

Authors: Yuyao Zhu, Sunmian Xu, Cheng Li, Hai Chen, and Jingxiang Wu

Supplementary Methods

Cross-cohort variable harmonization

Local laboratory values were converted from g/L to g/dL for hemoglobin and albumin and from micromoles/L to mg/dL for creatinine. VitalDB variables retained their source units. Procedure names were restricted to wedge resection, segmentectomy, or lobectomy. Surgical approach was mapped to VATS, open, or robotic categories. All local patients underwent arterial catheterization. The anesthesia information system stored invasive MAP every 5 min from anesthesia start to anesthesia end. Anesthesiologists reviewed the anesthesia record and corrected artifacts at the corresponding intraoperative time points before export; no additional research-stage numeric artifact filter was applied. Missing scheduled entries were completed before calculation, leaving no missing exported MAP values. For MAP_i at each valid 5-min time point, T_valid was the actual monitored time in minutes calculated from the anesthesia-start and anesthesia-end timestamps; mean MAP was mean(MAP_i), TWA deficit was sum[max(65 − MAP_i, 0) × 5]/T_valid, low-MAP fraction was sum[I(MAP_i < 65) × 5]/T_valid, and the longest episode was the maximum number of consecutive MAP_i values <65 mmHg multiplied by 5 min. Supplementary Code S1 implements these calculations. VitalDB used different sampling, filtering, integration, and window rules; additionally, its low-MAP fraction used surgical duration rather than valid monitored time. Table S6 therefore reports observed balance without claiming computational equivalence. The local postoperative-LOS time origin also remained unavailable for audit.

Supplementary Code S1: Local invasive-BP summary generation

`Supplementary_Code_S1_local_BP_summaries.py` calculates the four local BP summaries from a long-format, patient-level series of 5-min invasive MAP records. The code contains no patient data or institution-specific file paths. Required input fields, validation checks, formulas, and output names are documented in the file header.

Flexible calibration, risk distributions, and decision curves

Calibration intercept and slope were estimated from the model linear predictor. Flexible calibration curves used logistic regression with spline terms for predicted log odds. Pointwise 95% bands were obtained from 1000 bootstrap resamples. Predicted-risk histograms used 30 equal-width bins and were normalized within outcome groups. Decision curves were evaluated from threshold probability 0.05 to 0.70; model, treat-all, and treat-none net benefit were calculated in each bootstrap resample. No threshold-linked clinical action was prespecified, so the curves are descriptive.

Missing-data sensitivity analyses

The complete-case analysis applied the unchanged submitted models to external patients with complete values for all model predictors. The alternative-imputation analysis refitted both models in the local development cohort using iterative numeric imputation without missingness indicators; categorical most-frequent imputation and full-level one-hot encoding were retained. The refitted pipelines were applied unchanged to the 624-patient external paired sample.

Post hoc sample-size context

Development-sample context was evaluated using `pmsampsize` 1.1.3 with outcome prevalence 249/536, 26 or 31 transformed slope terms, temporal-validation AUROCs of 0.719 or 0.725, target global shrinkage 0.90, and random seed 20260715. External-validation precision was evaluated using `pmvalsampsize` 0.1.0 with prevalence 311/624, observed external discrimination,

calibration slope, observed-to-expected ratio, and linear-predictor distribution. Target 95% CI widths were 0.10 for AUROC and 0.20 for observed-to-expected ratio and calibration slope. These are post hoc context calculations, not an a priori design.

BP-feature correlation and coefficient stability

Spearman correlations among the four untransformed BP summaries were calculated separately in development, temporal-validation, and external cohorts. Coefficient stability was assessed in 300 bootstrap samples of the development cohort. Each bootstrap refit used the frozen preprocessing structure and C = 0.10. Intervals describe resampling variability of penalized, coding-dependent coefficients and are not causal confidence intervals.

Descriptive SHAP analysis

Exact linear SHAP values were calculated on the model log-odds scale using the mean transformed development vector as the reference. Contributions from encoded levels and missingness indicators were grouped to the source predictor. These values describe the fitted model only; they do not establish causal or independent predictor effects and do not identify why external performance changed.

Exploratory ICU outcome

ICU use was prespecified as exploratory. Only 18 temporal-validation ICU events were available, and prevalence differed markedly between centers. Results are therefore provided without comparative clinical inference.

Supplementary Tables

TABLE S1: Predictor missingness

Variable	Local development	Local temporal	VitalDB Korea
age	0 (0.0%)	0 (0.0%)	0 (0.0%)
sex	0 (0.0%)	0 (0.0%)	0 (0.0%)
bmi	0 (0.0%)	0 (0.0%)	0 (0.0%)
asa	0 (0.0%)	0 (0.0%)	15 (2.4%)
diabetes	0 (0.0%)	0 (0.0%)	0 (0.0%)
hypertension	0 (0.0%)	0 (0.0%)	0 (0.0%)
hb_g_dl	24 (4.5%)	3 (1.6%)	70 (11.2%)
alb_g_dl	24 (4.5%)	3 (1.6%)	84 (13.4%)
cr_mg_dl	24 (4.5%)	3 (1.6%)	83 (13.3%)
procedure_type	0 (0.0%)	0 (0.0%)	0 (0.0%)
surgery_approach	0 (0.0%)	0 (0.0%)	0 (0.0%)
surg_time	0 (0.0%)	0 (0.0%)	0 (0.0%)
blood_loss	0 (0.0%)	0 (0.0%)	210 (33.5%)
map_mean	9 (1.7%)	3 (1.6%)	2 (0.3%)
twa_map_lt65	0 (0.0%)	0 (0.0%)	2 (0.3%)
hypotension_fraction_lt65	0 (0.0%)	0 (0.0%)	2 (0.3%)
longest_map_lt65_min	0 (0.0%)	0 (0.0%)	2 (0.3%)

TABLE S2: Final model coefficients

Model	Encoded feature	Coefficient	Selected C
Baseline model	Intercept	0.01437	0.1
Baseline model	age	0.10198	0.1
Baseline model	bmi	0.172328	0.1
Baseline model	hb_g_dl	-0.005065	0.1
Baseline model	alb_g_dl	-0.00186	0.1
Baseline model	cr_mg_dl	0.043206	0.1
Baseline model	log_surg_time	0.105119	0.1
Baseline model	log_blood_loss	0.295561	0.1
Baseline model	missing: hb_g_dl	0.010147	0.1

Baseline model	missing: alb_g_dl	0.010147	0.1
Baseline model	missing: cr_mg_dl	0.010147	0.1
Baseline model	sex_0	0.102653	0.1
Baseline model	sex_1	-0.088282	0.1
Baseline model	asa_1.0	0.048403	0.1
Baseline model	asa_2.0	0.144988	0.1
Baseline model	asa_3.0	-0.17134	0.1
Baseline model	asa_4.0	-0.007681	0.1
Baseline model	diabetes_0	-0.008147	0.1
Baseline model	diabetes_1	0.022517	0.1
Baseline model	hypertension_0	-0.063482	0.1
Baseline model	hypertension_1	0.077853	0.1
Baseline model	procedure_type_lobectomy	0.485675	0.1
Baseline model	procedure_type_segmentectomy	0.33849	0.1
Baseline model	procedure_type_wedge_resection	-0.809795	0.1
Baseline model	surgery_approach_Open	0.417793	0.1
Baseline model	surgery_approach_Robotic	-0.344613	0.1
Baseline model	surgery_approach_VATS	-0.058809	0.1
BP-enhanced model	Intercept	0.020182	0.1
BP-enhanced model	age	0.115403	0.1
BP-enhanced model	bmi	0.172411	0.1
BP-enhanced model	hb_g_dl	0.00201	0.1
BP-enhanced model	alb_g_dl	0.003276	0.1
BP-enhanced model	cr_mg_dl	0.047842	0.1
BP-enhanced model	log_surg_time	0.09985	0.1
BP-enhanced model	log_blood_loss	0.309803	0.1
BP-enhanced model	map_mean	0.175197	0.1
BP-enhanced model	log_twa_map_lt65	-0.006732	0.1
BP-enhanced model	hypotension_fraction_lt65	-0.008311	0.1
BP-enhanced model	log_longest_map_lt65	0.136371	0.1
BP-enhanced model	missing: hb_g_dl	0.006236	0.1
BP-enhanced model	missing: alb_g_dl	0.006236	0.1
BP-enhanced model	missing: cr_mg_dl	0.006236	0.1
BP-enhanced model	missing: map_mean	-0.303685	0.1
BP-enhanced model	sex_0	0.109939	0.1
BP-enhanced model	sex_1	-0.089756	0.1
BP-enhanced model	asa_1.0	0.018365	0.1
BP-enhanced model	asa_2.0	0.143199	0.1
BP-enhanced model	asa_3.0	-0.142127	0.1
BP-enhanced model	asa_4.0	0.000746	0.1
BP-enhanced model	diabetes_0	0.007466	0.1
BP-enhanced model	diabetes_1	0.012717	0.1
BP-enhanced model	hypertension_0	-0.071031	0.1
BP-enhanced model	hypertension_1	0.091214	0.1
BP-enhanced model	procedure_type_lobectomy	0.480551	0.1
BP-enhanced model	procedure_type_segmentectomy	0.33241	0.1
BP-enhanced model	procedure_type_wedge_resection	-0.792778	0.1
BP-enhanced model	surgery_approach_Open	0.419878	0.1
BP-enhanced model	surgery_approach_Robotic	-0.346036	0.1
BP-enhanced model	surgery_approach_VATS	-0.053659	0.1

Coding note: All observed categorical levels were retained (`drop=None`); there was no omitted reference category. Coefficients are coding-dependent under L2 penalization. With `liblinear`, `fit_intercept=True`, and `intercept_scaling=1`, the intercept is represented as a synthetic feature and is regularized.

TABLE S3: Penalization selection

Model	Outer-fold selected C values	Final selected C
Baseline model	[0.1, 0.3, 0.1, 0.1, 0.3, 0.3, 0.3, 0.3, 0.3, 0.3]	0.1
BP-enhanced model	[0.1, 0.3, 0.1, 0.3, 0.1, 0.3, 0.3, 0.3, 0.3, 0.3]	0.1

TABLE S4: Exploratory ICU performance

Validation	Model	n/events	AUROC (95% CI)	Average precision (95% CI)	Brier score (95% CI)	Calibration intercept	Calibration slope
Nested internal validation	Baseline model	536/68	0.574 (0.500-0.650)	0.153 (0.115-0.216)	0.111 (0.092-0.133)	-0.225	0.691
Local temporal validation (2025)	Baseline model	184/18	0.590 (0.472-0.690)	0.116 (0.069-0.188)	0.092 (0.062-0.121)	-0.397	0.394

VitalDB Korea external validation	Baseline model	624/378	0.624 (0.582-0.669)	0.724 (0.676-0.774)	0.459 (0.430-0.487)	2.424	0.999
Nested internal validation	BP-enhanced model	536/68	0.573 (0.497-0.654)	0.163 (0.118-0.229)	0.111 (0.092-0.133)	-0.191	0.346
Local temporal validation (2025)	BP-enhanced model	184/18	0.683 (0.553-0.795)	0.173 (0.101-0.331)	0.089 (0.060-0.116)	-0.433	1.051
VitalDB Korea external validation	BP-enhanced model	624/378	0.589 (0.540-0.633)	0.684 (0.631-0.739)	0.441 (0.414-0.468)	2.255	0.618

TABLE S5: Operation-window fixed-interval VitalDB sensitivity

scenario	metric	estimate (95% CI)
1min_bin_median	auroc	-0.008 (-0.022 to 0.008)
1min_bin_median	average_precision	-0.014 (-0.032 to 0.003)
1min_bin_median	brier_score	0.005 (0.000 to 0.010)
1min_bin_median	calibration_intercept	-0.197 (-0.213 to -0.182)
1min_bin_median	calibration_slope	-0.030 (-0.090 to 0.035)
2min_bin_median	auroc	-0.008 (-0.023 to 0.008)
2min_bin_median	average_precision	-0.016 (-0.034 to 0.000)
2min_bin_median	brier_score	0.005 (0.000 to 0.010)
2min_bin_median	calibration_intercept	-0.197 (-0.213 to -0.182)
2min_bin_median	calibration_slope	-0.027 (-0.087 to 0.034)
5min_bin_median	auroc	-0.007 (-0.023 to 0.008)
5min_bin_median	average_precision	-0.016 (-0.033 to 0.002)
5min_bin_median	brier_score	0.005 (-0.000 to 0.010)
5min_bin_median	calibration_intercept	-0.194 (-0.209 to -0.179)
5min_bin_median	calibration_slope	-0.030 (-0.091 to 0.033)

TABLE S6: Predictor standardized mean differences

variable	level	smd_temporal_vs_development	smd_external_vs_development
age	continuous	-0.007589127334997	0.2236788229667353
bmi	continuous	-0.0581959485884398	-0.0578610267245246
hb_g_dl	continuous	-0.0972347676237547	-0.4222386823914275
alb_g_dl	continuous	-0.0629644048714701	-0.3061757973135006
cr_mg_dl	continuous	-0.091693323678684	0.2990298566479151
surg_time	continuous	-0.0152430704687007	0.2799178178251921
blood_loss	continuous	-0.0775025102529359	0.2236768244282762
map_mean	continuous	0.0672479683221961	0.9742723439736024
twa_map_lt65	continuous	0.0418665302361392	0.9003597522161993
hypotension_fraction_lt65	continuous	-0.0437934027161657	0.6938645374417554
longest_map_lt65_min	continuous	-0.1068170923444946	0.4479729801441434
sex	1 vs 0	0.0853866812276876	-0.0272998029413978
diabetes	1 vs 0	-0.0344598385552501	0.0644898807254411
hypertension	1 vs 0	-0.0768752069984115	0.1859059688242607
asa	1.0	0.0617945355085297	-0.9577468446805882
asa	2.0	-0.1115754507827433	0.8411229978222823
asa	3.0	0.083929136343709	0.0469827816899806
asa	4.0	-0.0454983252644248	-0.1050283868945155
procedure_type	lobectomy	0.0357160416231148	0.9265592326631522
procedure_type	segmentectomy	-0.0388293601985331	-0.7472693223490617
procedure_type	wedge_resection	0.0131750954775018	-0.187154519269544
surgery_approach	Open	-0.0125703083613251	-0.385726206219295
surgery_approach	Robotic	-0.1393681691304971	-0.3940855882609927
surgery_approach	VATS	0.0916025950854408	0.5662983030948597

TABLE S7: VitalDB ART_MBP quality-control audit

QC item	Value
procedure_restricted_cases	626.0
track_files_available	625.0
required_columns_available	625.0
feature_summary_eligible	624.0
feature_summary_ineligible	2.0
raw_rows	3746159.0
missing_or_nonnumeric_rows	0.0
map_outside_20_200_rows	305456.0
valid_rows_after_filter	3440703.0

valid_rows_within_operation_window	2593982.0
intervals_gt_30_seconds	898.0
cases_with_interval_gt_30_seconds	417.0
nonpositive_intervals	11134.0
cases_using_default_2_second_interval	0.0
fraction_valid_rows_within_operation_window	0.7539104653903578

TABLE S8: Development-set preprocessing parameters

model	component	feature	imputation_value	robust_center	robust_scale	learned_categories
baseline_model	numeric	age	60.0	60.0	16.0	
baseline_model	numeric	bmi	23.7	23.7	4.400000000000002	
baseline_model	numeric	hb_g_dl	13.690000000000001	13.69	1.462500000000002	
baseline_model	numeric	alb_g_dl	4.2700000000000005	4.2700000000000005	0.33	
baseline_model	numeric	cr_mg_dl	0.7630090497737556	0.7630090497737556	0.2135180995475112	
baseline_model	numeric	log_surg_time	4.795790545596741	4.795790545596741	0.4979497710492166	
baseline_model	numeric	log_blood_loss	4.61512051684126	4.61512051684126	1.201014292593645	
baseline_model	numeric	missing:hb_g_dl		0.0	1.0	
baseline_model	numeric	missing:alb_g_dl		0.0	1.0	
baseline_model	numeric	missing:cr_mg_dl		0.0	1.0	
baseline_model	categorical	sex	1			["0", "1"]
baseline_model	categorical	asa	1.0			["1.0", "2.0", "3.0", "4.0"]
baseline_model	categorical	diabetes	0			["0", "1"]
baseline_model	categorical	hypertension	0			["0", "1"]
baseline_model	categorical	procedure_type	wedge_resection			["lobectomy", "segmentectomy", "wedge_resection"]
baseline_model	categorical	surgery_approach	VATS			["Open", "Robotic", "VATS"]
bp_enhanced_model	numeric	age	60.0	60.0	16.0	
bp_enhanced_model	numeric	bmi	23.7	23.7	4.400000000000002	
bp_enhanced_model	numeric	hb_g_dl	13.690000000000001	13.69	1.462500000000002	
bp_enhanced_model	numeric	alb_g_dl	4.2700000000000005	4.2700000000000005	0.33	
bp_enhanced_model	numeric	cr_mg_dl	0.7630090497737556	0.7630090497737556	0.2135180995475112	
bp_enhanced_model	numeric	log_surg_time	4.795790545596741	4.795790545596741	0.4979497710492166	
bp_enhanced_model	numeric	log_blood_loss	4.61512051684126	4.61512051684126	1.201014292593645	
bp_enhanced_model	numeric	map_mean	80.2	80.2	6.949999999999989	
bp_enhanced_model	numeric	log_twa_map_lt65	0.0	0.0	0.009950330853168	
bp_enhanced_model	numeric	hypotension_fraction_lt65	0.0	0.0	1.0	
bp_enhanced_model	numeric	log_longest_map_lt65	0.0	0.0	1.0	
bp_enhanced_model	numeric	missing:hb_g_dl		0.0	1.0	
bp_enhanced_model	numeric	missing:alb_g_dl		0.0	1.0	
bp_enhanced_model	numeric	missing:cr_mg_dl		0.0	1.0	
bp_enhanced_model	numeric	missing:map_mean		0.0	1.0	
bp_enhanced_model	categorical	sex	1			["0", "1"]
bp_enhanced_model	categorical	asa	1.0			["1.0", "2.0", "3.0", "4.0"]
bp_enhanced_model	categorical	diabetes	0			["0", "1"]
bp_enhanced_model	categorical	hypertension	0			["0", "1"]
bp_enhanced_model	categorical	procedure_type	wedge_resection			["lobectomy", "segmentectomy", "wedge_resection"]
bp_enhanced_model	categorical	surgery_approach	VATS			["Open", "Robotic", "VATS"]

TABLE S9: Post hoc sample-size context

analysis	model	observed_n	observed_events	parameters	cstatistic_input	prevalence_input	calibration_slope_input	oe_input	target_ci_width	recommended_n	recommended_events	limiting_criterion
post_hoc_development_context	baseline	536	249	26.0	0.71941	0.46455223880597				1484	690	global_shrinkage_0.90
post_hoc_development_context	bp_enhanced	536	249	31.0	0.72543	0.46455223880597				1673	778	global_shrinkage_0.90
post_hoc_external_validation_precision_context	baseline	624	311		0.62252	0.498397435897436	0.569969	0.951134		3790	1889	calibration_slope_CI_width_0.20
post_hoc_external_validation_precision_context	bp_enhanced	624	311		0.615463	0.498397435897436	0.533772	0.910783		3563	1776	calibration_slope_CI_width_0.20

TABLE S10: Missing-data sensitivity analyses

strategy	model	n	events	event_rate	auroc	average_precision	brier_score	calibration_intercept	calibration_slope	mean_predicted_risk	auroc_ci_low	auroc_ci_high	average_precision_ci_low	average_precision_ci_high	brier_score_ci_low	brier_score_ci_high	calibration_intercept_ci_low	calibration_intercept_ci_high	calibration_slope_ci_low	calibration_slope_ci_high
complete_case_unchanged_submitted_model	baseline_model	356	200	0.5617977528089888	0.6019871794871795	0.6703072356988791	0.2462656797130309	0.0227181002085 89	0.4867093429412774	0.5568535080412 29	0.5419355562994589	0.6587824494346233	0.6078280431085904	0.7379749659778342	0.2296742521301333	0.2639483563051293	-0.2099474211060711	0.262965069234342	0.2036002899645839	0.778706904338285
complete_case_unchanged_submitted_model	bp_enhanced_model	356	200	0.5617977528089888	0.6049679487179487	0.6652347027456442	0.2450618988813242	-0.0839636573136676	0.5014251637177712	0.5797849871540599	0.5421232455932169	0.6611228923941895	0.5970946619667935	0.7359648575997384	0.2274623286663504	0.2644222517890602	-0.3205298454547919	0.1545802045495693	0.2259210353773925	0.7830406659756202
iterative_numeric_imputation_no_missing_indicators	baseline_model	624	311	0.4983974358974359	0.6218115324163012	0.6234015390045623	0.2453744193720 81	-0.1027716578866819	0.5688554523528 18	0.5209262860175465	0.5773553298309392	0.6656266229516338	0.5702603674815516	0.6828860152407352	0.2319798008389516	0.2591223411197173	-0.2763174816016865	0.0702924572920381	0.3536860848754736	0.7942240470893555
iterative_numeric_imputation_no_missing_indicators	bp_enhanced_model	624	311	0.4983974358974359	0.6130692499717494	0.6106418429084608	0.2490374372261968	-0.2162908877587511	0.5229223043324317	0.5452874066124923	0.5693321789835402	0.6585981164921646	0.556547626171521	0.6746626117126096	0.2345042773658 84	0.2637248258946448	-0.3920998803853836	-0.0478195814177555	0.3189236576964085	0.7389646974416851

TABLE S11A: BP-feature Spearman correlations

cohort	feature_1	feature_2	n	spearman_rho	p_value
local_development	map_mean	log_twa_map_lt65	527	0.0028662617825028	0.9476619131655948
local_development	map_mean	hypotension_fraction_lt65	527	-0.0412626503351888	0.3444553849841091
local_development	map_mean	log_longest_map_lt65	527	-0.0422825798033449	0.3326511345271276
local_development	log_twa_map_lt65	hypotension_fraction_lt65	536	0.8112902604370282	1.4068963660486472e-126
local_development	log_twa_map_lt65	log_longest_map_lt65	536	0.8042277189305744	9.109762223830794e-123
local_development	hypotension_fraction_lt65	log_longest_map_lt65	536	0.9546534218960824	3.72439358545468e-283
local_temporal	map_mean	log_twa_map_lt65	181	0.0407497818988983	0.5859871478675716
local_temporal	map_mean	hypotension_fraction_lt65	181	-0.0399894511976107	0.5930028684107214
local_temporal	map_mean	log_longest_map_lt65	181	-0.0161426329981338	0.8292322484408291
local_temporal	log_twa_map_lt65	hypotension_fraction_lt65	184	0.6916453997452653	1.616686234745172e-27
local_temporal	log_twa_map_lt65	log_longest_map_lt65	184	0.7224563319503701	5.533486123674815e-31
local_temporal	hypotension_fraction_lt65	log_longest_map_lt65	184	0.962861175563739	1.9718454739811593e-105
vitaldb_external	map_mean	log_twa_map_lt65	624	-0.4831979256698358	8.039956907555839e-38
vitaldb_external	map_mean	hypotension_fraction_lt65	624	-0.7057636745277195	3.5222820472831803e-95
vitaldb_external	map_mean	log_longest_map_lt65	624	-0.6162505578359021	1.5813863909056412e-66
vitaldb_external	log_twa_map_lt65	hypotension_fraction_lt65	624	0.8253788862822571	1.44801296903463e-156
vitaldb_external	log_twa_map_lt65	log_longest_map_lt65	624	0.7775703070804977	1.9353367805014934e-127
vitaldb_external	hypotension_fraction_lt65	log_longest_map_lt65	624	0.8914048369300009	5.910898413604539e-216

TABLE S11B: Bootstrap coefficient stability

feature	reference_coefficient	bootstrap_n	bootstrap_median	bootstrap_q1	bootstrap_q3	bootstrap_ci_low	bootstrap_ci_high	same_sign_fraction
numeric__age	0.115402995247831	300	0.1059419498412346	0.0174652933539721	0.1858158740941435	-0.1367986991490364	0.3394993158093206	0.8166666666666667
numeric__bmi	0.1724109143666782	300	0.1596133181707651	0.0894566675004568	0.2328982885934322	-0.052207390108581	0.3553268855031039	0.9366666666666666
numeric__hb_g_dl	0.0020102833595509	300	-0.0040250572633148	-0.0688276872090753	0.0827274261326564	-0.2051955731896913	0.2083165640091644	0.48
numeric__alb_g_dl	0.0032755572541683	300	0.0066507343049162	-0.0634090191949626	0.0650938782056556	-0.209949703673076	0.2077690426399068	0.5166666666666667
numeric__cr_mg_dl	0.0478421450439905	300	0.0617761260245062	-0.0225981357436082	0.1223203973973663	-0.1697111362619358	0.2653590389210666	0.69

numeric__log_surg_time	0.0998498146369353	300	0.0972399233527977	0.0360523724469805	0.1658814578884717	-0.109287397614628	0.3402686360396133	0.8433333333333334
numeric__log_blood_loss	0.3098029406312451	300	0.3116864121789103	0.2362241219239855	0.4003384394210676	0.1049692174007131	0.5829084765517963	1.0
numeric__map_mean	0.1751972411531699	300	0.1765972572246059	0.1003033276708501	0.2437518084249761	-0.0381331546675343	0.3625952309215751	0.9533333333333334
numeric__log_twa_map_lt65	-0.0067320812651923	300	-0.0095836523587173	-0.0163627080869163	-0.0045350764994666	-0.0322423660066132	0.0054796017204501	0.9033333333333332
numeric__hypotension_fraction_lt65	-0.0083108127153107	300	-0.0080709979746674	-0.016499527602251	0.0013584121219796	-0.0357507949853247	0.0167742450480978	0.7166666666666667
numeric__log_longest_map_lt65	0.1363706796679843	300	0.1557396853542985	0.0641298154982537	0.2387378473974736	-0.0642091471855595	0.3679908518624345	0.9033333333333332
numeric__missingindicator_hb_g_dl	0.0062358522372726	300	0.00846957521492	-0.0438442140841318	0.0731698893060015	-0.1552388381353028	0.1782150188488517	0.56
numeric__missingindicator_alb_g_dl	0.0062358522372726	300	0.00846957521492	-0.0438442140841318	0.0731698893060015	-0.1552388381353028	0.1782150188488517	0.56
numeric__missingindicator_cr_mg_dl	0.0062358522372726	300	0.00846957521492	-0.0438442140841318	0.0731698893060015	-0.1552388381353028	0.1782150188488517	0.56
numeric__missingindicator_map_mean	-0.3036850585750029	300	-0.2902402569648926	-0.3798146165464507	-0.1854313989595978	-0.5387582711648179	-0.0308872691639512	0.9866666666666668
categorical__sex_0	0.1099386185669293	300	0.0998897602759194	0.0387054319295847	0.1633508030564056	-0.0610220101569288	0.2747980249647439	0.8766666666666667
categorical__sex_1	-0.0897561186157751	300	-0.0810362387861805	-0.1424510536721688	-0.0231888899983777	-0.2569230242116697	0.0756308153609016	0.8466666666666667
categorical__asa_1.0	0.0183645403199272	300	0.0136231824989486	-0.0468153823400687	0.0678437206968467	-0.1735490695144346	0.171643262678748	0.55
categorical__asa_2.0	0.1431989811254334	300	0.1417301658292789	0.0542060957176959	0.2264131488115244	-0.0631355509841386	0.3329626294916823	0.8866666666666667
categorical__asa_3.0	-0.1421269694459343	300	-0.1217544755085886	-0.2141067401766287	-0.0491419664734814	-0.3580316478874156	0.0935138949775963	0.85
categorical__asa_4.0	0.0007459479517315	300	0.0033012761702732	-0.057142342375589	0.0643966947504418	-0.1670824763811472	0.1749640794300467	0.5066666666666667
categorical__diabetes_0	0.0074657670047233	300	0.0062397886885787	-0.0497561155410819	0.0745320602504417	-0.1603361150783065	0.2080623491088213	0.52
categorical__diabetes_1	0.0127167329464332	300	0.0154935462957149	-0.0653735054574396	0.0812854719129013	-0.2171155403118684	0.2055470536324541	0.5566666666666666
categorical__hypertension_0	-0.0710314443646988	300	-0.0804503422739253	-0.1388792748538168	-0.0232943847383582	-0.2452217117302585	0.1018958356530765	0.8333333333333334
categorical__hypertension_1	0.0912139443158609	300	0.1048493689983697	0.0384657665033396	0.1541101462386289	-0.107237871969275	0.2817781929230041	0.8733333333333333
categorical__procedure_type_lobectomy	0.4805508199226592	300	0.468582669202311	0.4055108610072786	0.5567750721354303	0.2720291736399033	0.708321917061499	1.0
categorical__procedure_type_segmentectomy	0.3324095686008971	300	0.3361630303781213	0.2697026011374933	0.4173641427273128	0.1153313020604444	0.5406262114648664	1.0
categorical__procedure_type_wedge_resection	-0.7927778885723972	300	-0.8002142252646309	-0.8713603120741904	-0.7286004908571226	-0.977622880909974	-0.5831066275130659	1.0
categorical__surgery_approach_Open	0.419878045040027	300	0.419942471792089	0.3536206742377658	0.4962573980920804	0.180949728289294	0.6353379401259284	1.0
categorical__surgery_approach_Robotic	-0.3460362831044709	300	-0.3375149986758499	-0.4189922468735637	-0.2396639419795354	-0.5836005095370541	-0.0926716693846835	0.9933333333333332
categorical__surgery_approach_VATS	-0.0536592619843972	300	-0.0737004816550676	-0.1353864488489743	-0.0002972681843775	-0.2442387457750595	0.1608510157267013	0.7533333333333333

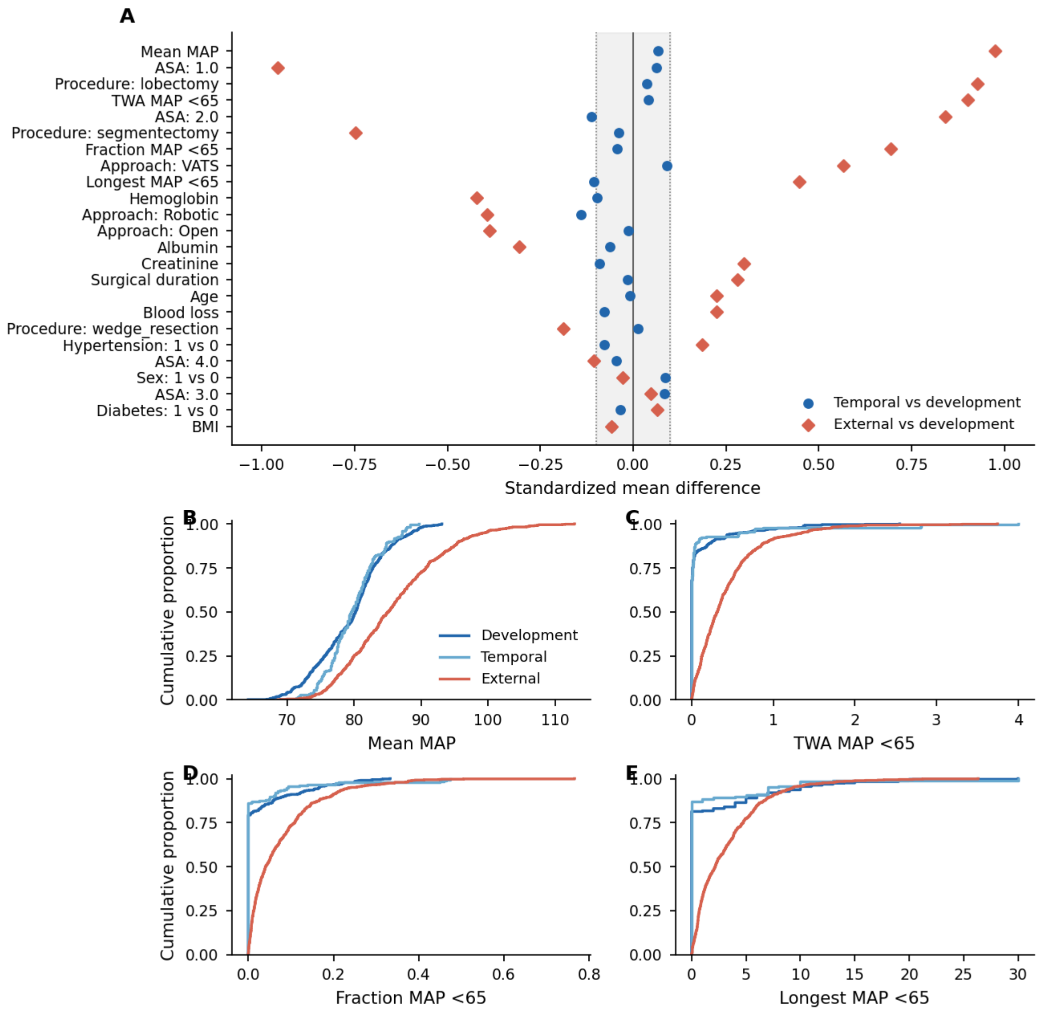

Figure S1. Predictor shift and overlap. (**A**), Standardized mean differences comparing local temporal validation and VitalDB external validation with local development; the shaded region denotes absolute SMD < 0.10. (**B**–**E**), Empirical cumulative distributions of the four BP summaries. Positive and negative SMD signs indicate direction only; magnitude reflects imbalance. BP, blood pressure; MAP, mean arterial pressure; TWA, time-weighted average; VATS, video-assisted thoracoscopic surgery.

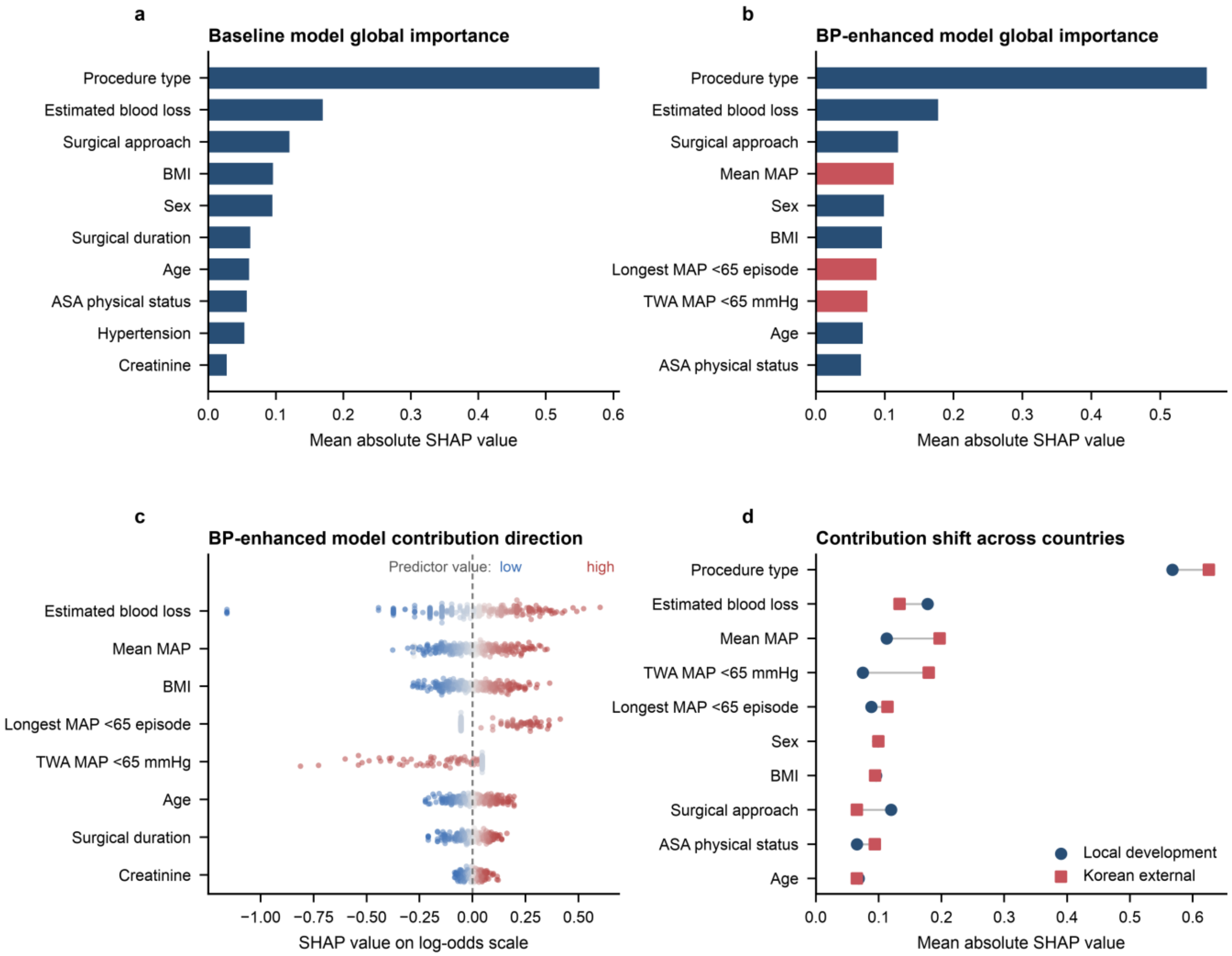

Figure S2. Descriptive model contributions for PLOS. (**a**,**b**), Mean absolute linear SHAP values in local development for the baseline and BP-enhanced models. (**c**), Patient-level contribution distributions for selected numeric predictors. (**d**), Contributions from the unchanged BP-enhanced model in local development and VitalDB. Values are on the fitted model log-odds scale and are descriptive; they do not represent causal effects, independent predictor effects, or explanations for transportability failure. SHAP, Shapley additive explanations; BP, blood pressure; MAP, mean arterial pressure; TWA, time-weighted average.
